# MUC1 aptamer-tethered H40-TEPA-PEG nanoconjugates for targeted siRNA-delivery and gene silencing in breast cancer cells

**DOI:** 10.3389/fbioe.2024.1383495

**Published:** 2024-04-18

**Authors:** Rajesh Salve, Niladri Haldar, Aazam Shaikh, Rajkumar Samanta, Devyani Sengar, Surajit Patra, Virendra Gajbhiye

**Affiliations:** ^1^ Nanobioscience Group, Agharkar Research Institute, Pune, India; ^2^ Savitribai Phule Pune University, Pune, India

**Keywords:** mucin-1, bis-MPA polyester, siRNA, survivin, breast cancer

## Abstract

With a prevalence of 12.5% of all new cancer cases annually, breast cancer stands as the most common form of cancer worldwide. The current therapies utilized for breast cancer are constrained and ineffective in addressing the condition. siRNA-based gene silencing is a promising method for treating breast cancer. We have developed an aptamer-conjugated dendritic multilayered nanoconjugate to treat breast cancer. Initially, we transformed the hydroxyl groups of the hyperbranched bis-MPA polyester dendrimer into carboxylic groups. Subsequently, we linked these carboxylic groups to tetraethylenepentamine to form a positively charged dendrimer. In addition, the mucin-1 (MUC1) aptamer was attached to the dendrimer using a heterobifunctional polyethylene glycol. Characterizing dendrimers involved ^1^H NMR and dynamic light scattering techniques at every production stage. A gel retardation experiment was conducted to evaluate the successful binding of siRNA with targeted and non-targeted dendrimers. The targeted dendrimers exhibited no harmful effects on the NIH-3T3 fibroblast cells and RBCs, indicating their biocompatible characteristics. Confocal microscopy demonstrated significant higher uptake of targeted dendrimers than non-targeted dendrimers in MCF-7 breast cancer cells. The real-time PCR results demonstrated that the targeted dendrimers exhibited the most pronounced inhibition of the target gene expression compared to the non-targeted dendrimers and lipofectamine-2000. The caspase activation study confirmed the functional effect of survivin silencing by dendrimer, which led to the induction of apoptosis in breast cancer cells. The findings indicated that Mucin-1 targeted hyperbranched bis-MPA polyester dendrimer carrying siRNA could successfully suppress the expression of the target gene in breast cancer cells.

## 1 Introduction

During the year 2020, breast cancer emerged as the most often detected form of cancer globally. The number of newly diagnosed breast cancer cases surpassed 2.26 million, resulting in about 685,000 deaths globally. Among females, breast cancer ranked as the leading cause of cancer mortality, and overall, it ranked as the fifth most prevalent cause of cancer-related deaths. The heterogeneity of breast cancer is characterized by the variances in the morphology of the tumor cells, genetic differences, and molecular level changes in several associated indicators (Moudgil et al., 2023). The treatment regime for breast cancer involves chemotherapeutic drugs and surgery, but these have certain limitations which make them ineffective (Moo et al., 2018). Several genes are involved in mechanisms like membrane transporter activity, dysregulation of apoptosis, etc., which can be regulated to treat breast cancer. The inhibitor of apoptosis protein family (IAPs) plays a major role in controlling apoptosis. Survivin, a member of this protein family, holds a crucial role due to its excessive presence in cancer cells. Its overexpression in tumor cells is believed to enhance tumor growth by many mechanisms, involving the alteration in apoptosis and cell division, altered response to anticancer medicines, and provoking survival of cancer stem cells (Garg et al., 2016). Because of its high expression, it is an excellent target for tumor diagnosis prognosis and cancer therapeutics; hence, regulating its expression becomes a crucial target for cancer treatment (Jaiswal et al., 2015).

Gene silencing by RNA interference (RNAi) could be one of the most promising treatments for breast cancer. RNAi is a process of gene silencing that occurs after transcription and is unique to specific sequences. There has been significant attention paid to small interfering RNA (siRNA) based on RNAi, which have great potential in cancer therapeutics. The primary obstacle in achieving siRNA-mediated targeted gene silencing is the requirement for efficient and secure delivery techniques (Tambe et al., 2017). Despite the considerable potential of siRNA delivery in biomedical applications, several limitations hinder its widespread use. There is a need for protective carriers for the siRNA delivery system due to its poor stability, lower cellular uptake, and rapid clearance.

Unlike microparticles, hydrogels, and implants, nanoparticles may be precisely designed to offer many capabilities, such as controlled cargo release, targeted cell interaction, and improved cellular absorption (Gavas et al., 2021). Various nanocarriers have been created to effectively transport nucleic acids to cancer cells. The nanocarriers inhibit the enzymatic breakdown of the siRNA and improve the internalization by cells (Tambe et al., 2023). Dendrimers are precisely engineered, highly branched, spherical, and monodisperse nanostructures. They are created by repeated reaction procedures, resulting in a distinct and accurate branching pattern. The changeable molecular weight, abundant terminal functional groups, and ability to encapsulate guest molecules in internal cavities make dendrimers very promising as drug-delivery vehicles (Kesharwani et al., 2012). Dendrimers have many uses, such as protein mimetics, drug delivery agents, diagnostics tools, gene delivery vehicles, etc. (Chis et al., 2020). The utilization of nanocarriers is mostly limited due to factors such as absorption by the reticuloendothelial system (RES), immunogenicity, stability issues, drug leakage, hemolytic toxicity, and hydrophobic nature. To overcome these limitations, polyethylene glycol (PEG) is conjugated to the nanocarriers, enhancing the biocompatibility of the nanocarriers. The efficacy of these nanocarriers can be improved by using ligands to bind to the tumor surface receptors. Among the various ligands aptamers are a group of oligonucleotide ligands that have been found for their ability to specifically identify cancer cells. They are employed in conjunction with nanocarriers at the molecular level, functioning as chemical antibodies. These molecules, known as single-stranded DNA or RNA, are very small (20–60 nucleotides) and are produced using SELEX technology. They have a strong ability to bind to different receptors with a high level of affinity and specificity (Narwade et al., 2023). Till date, no one tried combination of tetraethylenepentamine modified bis-MPA dendrimer, MUC1, PEG and survivin siRNA for breast cancer therapy.

Therefore, in this study, we have designed a targeted bis-MPA polyester dendrimer to deliver siRNA to breast cancer cells. This multifunctional degradable dendrimer was created by conjugating tetraethylenepentamine, succeeded by mucin-1 aptamer conjugation via a heterobifunctional polyethylene glycol linker. The characterization of dendrimers involved ^1^H NMR and dynamic light scattering techniques at every stage of production. A gel retardation experiment was conducted to evaluate the successful binding of siRNA with targeted and non-targeted dendrimers. The aptamer-conjugated dendrimers exhibited no harmful effects on the NIH-3T3 fibroblast cells and RBCs, indicating their biocompatible characteristics. The investigation on the cellular absorption of dendrimers in a breast cancer cell line demonstrated the successful internalization of targeted nanoconjugates in the MCF-7 cell line, as opposed to non-targeted dendrimers. The real-time PCR results demonstrated that the targeted dendrimers exhibited the most pronounced inhibition of the target gene expression compared to the non-targeted dendrimers and lipofectamine-2000. The caspase activation study confirmed the functional effect of survivin silencing by dendrimer, which led to the induction of apoptosis in breast cancer cells. This study demonstrated that, the designed bis-MPA polyester dendrimer platform was highly biocompatible, specific for MUC1 overexpressing cancer cells, and significant target gene silencing ability *in vitro.*

## 2 Results and discussion

### 2.1 Synthesis and characterization

**FIGURE 1:**
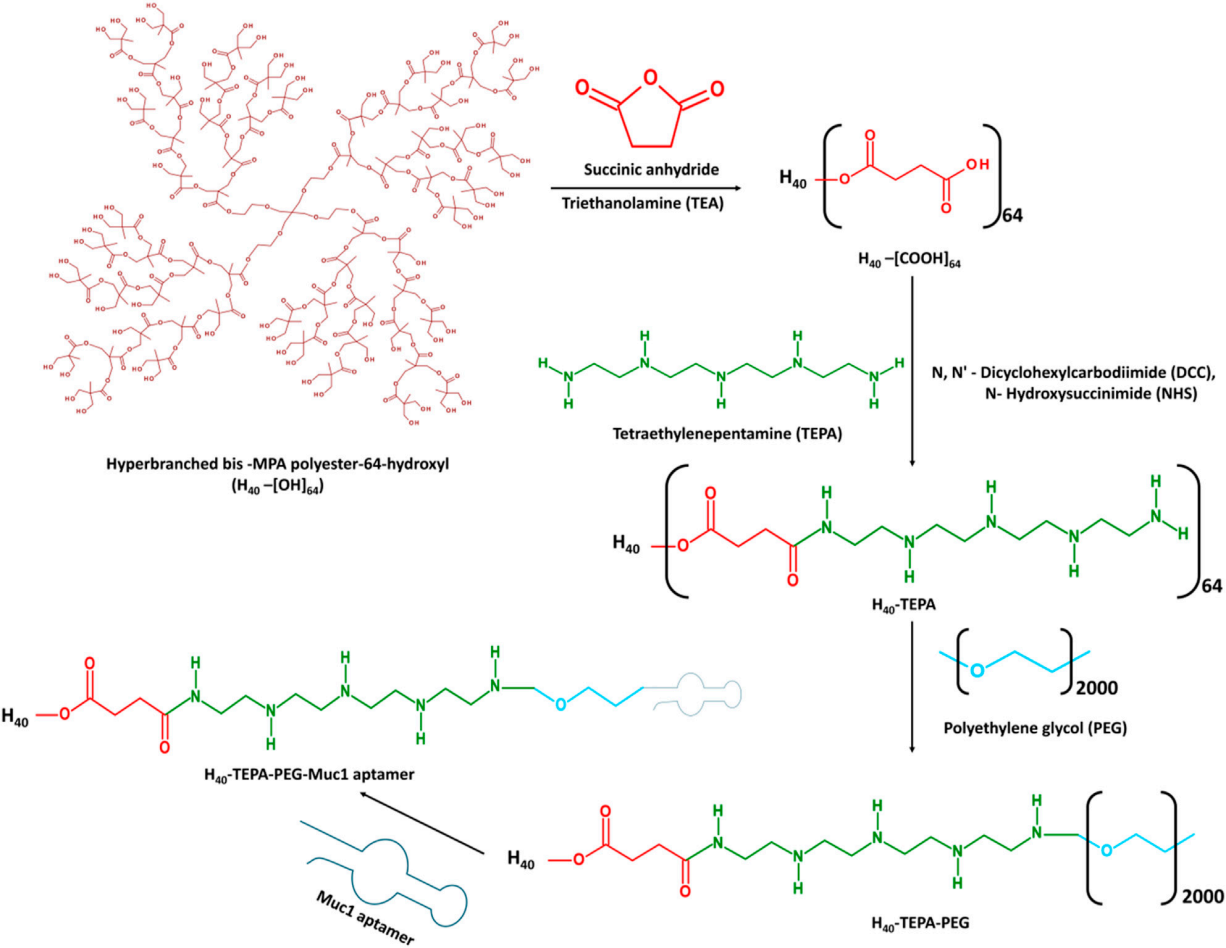
Synthesis scheme of MUC1 aptamer-conjugated hyperbranched bis-MPA polyester dendrimer.

**FIGURE 2:**
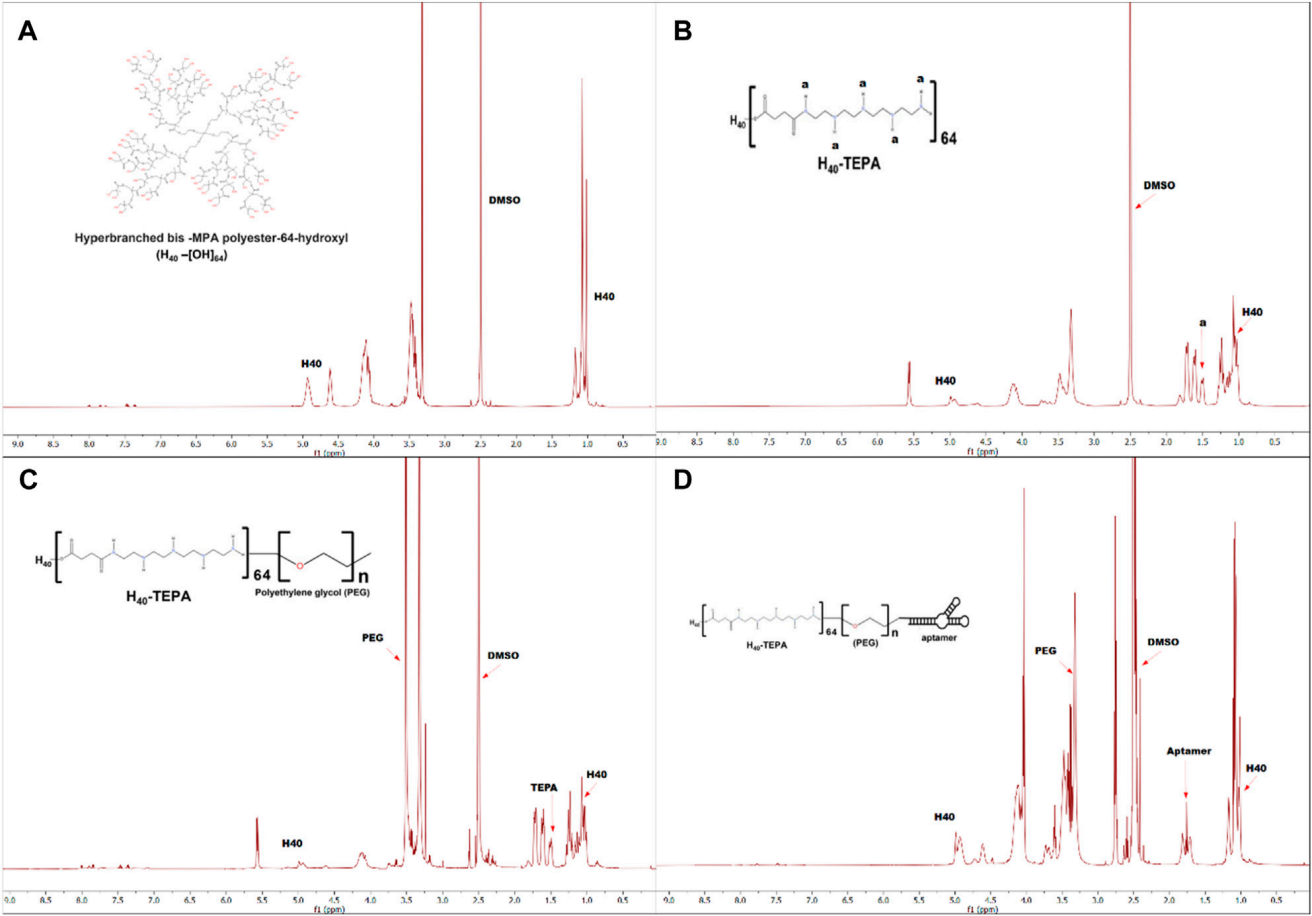
^1^H-NMR spectra of **(A)** H40-OH, **(B)** H40-TEPA, **(C)** H40-TEPA-PEG, **(D)** H40-TEPA-PEG-MUC1 in DMSO-d6.

Figure 1 shows schematic representation for synthesis of MUC1 aptamer-conjugated hyperbranched bis-MPA polyester dendrimer. All the intermediate and final H40 polymer products were recorded on Bruker Avance III HD NMR 500 MHz spectrometer using DMSO as a solvent (Figures 2A–D). Tetraethylenepentamine (TEPA) signature peaks could be observed at 1.5 ppm (amine groups), confirming the formation of H40-TEPA (Figure 2B). The dendrimers were further conjugated with heterobifunctional polyethylene glycol (PEG) to increase biocompatibility and solubility and protect siRNAs. The peak of PEG was observed at 3.5 ppm in the NMR spectra (Figure 2C). To target cancer cells, the nanoparticles were modified with aptamer depicting NMR peaks from 1.8 to 2.10 ppm (Figure 2D).

The hydrodynamic size of the dendrimers was measured by dynamic light scattering. The non-targeted and targeted dendrimer had sizes of 46.23 ± 2.14 and 68.42 ± 3.78 nm, respectively (Supplementary Figure S1). Zeta potential analysis showed a surface charge of 9.67 ± 2.33 and 3.62 ± 1.24 mV for non-targeted and targeted dendrimers, respectively (Supplementary Figure S1). An increase in size and reduction in the zeta potential of targeted dendrimers compared to non-targeted dendrimers was due to the conjugation of MUC1 aptamer. siRNA-loaded targeted and non-targted dendrimers were also characterized for size and surface charge. The hydrodynamic size of the siRNA-loaded targeted and non-targted dendrimers was found to be 76.41 ± 2.27 and 58.77 ± 3.38 nm, respectively (Supplementary Figure S2). Zeta potential analysis of siRNA-loaded dendrimers showed a surface charge of −5.09 ± 1.44 and −2.32 ± 1.65 mV for targeted and non-targeted dendrimers, respectively (Supplementary Figure S2).

### 2.2 Gel retardation assay

Nucleic acid molecules are effectively loaded onto cationic dendrimers, and the negative charge of siRNA can be neutralized, inhibiting the mobility of siRNA (Tambe et al., 2017). A gel retardation experiment was carried out to measure the dendrimers and siRNA interaction. The negatively charged siRNA molecules were complexed to targeted dendrimers, which reduced siRNA mobility in agarose gel electrophoresis. Different nitrogen-to-phosphate (N/P) ratios were used to investigate the potential of targeted dendrimers for the loading of siRNA in a stable manner. The agarose gel electrophoresis experiment revealed a gradual reduction in the movement of siRNA as the concentration of dendrimers increased. The complete complexation of the siRNA with targeted dendrimers and non-targeted dendrimers occurred at 25:1 and 20:1 N/P ratio, respectively (Supplementary Figure S3). The higher dendrimers to siRNA ratio for targeted dendrimers was due to the presence of aptamer.

### 2.3 Cell viability assay

To establish the toxicity level, NIH-3T3 cells were subjected to treatment with targeted dendrimers at five different concentrations, followed by an MTT experiment. The targeted dendrimers showed no toxicity at concentrations as high as 150 μg/mL (Supplementary Figure S4). When considerable concentrations of dendrimers are required, they must have a low cytotoxicity.

### 2.4 Cellular uptake

**FIGURE 3:**
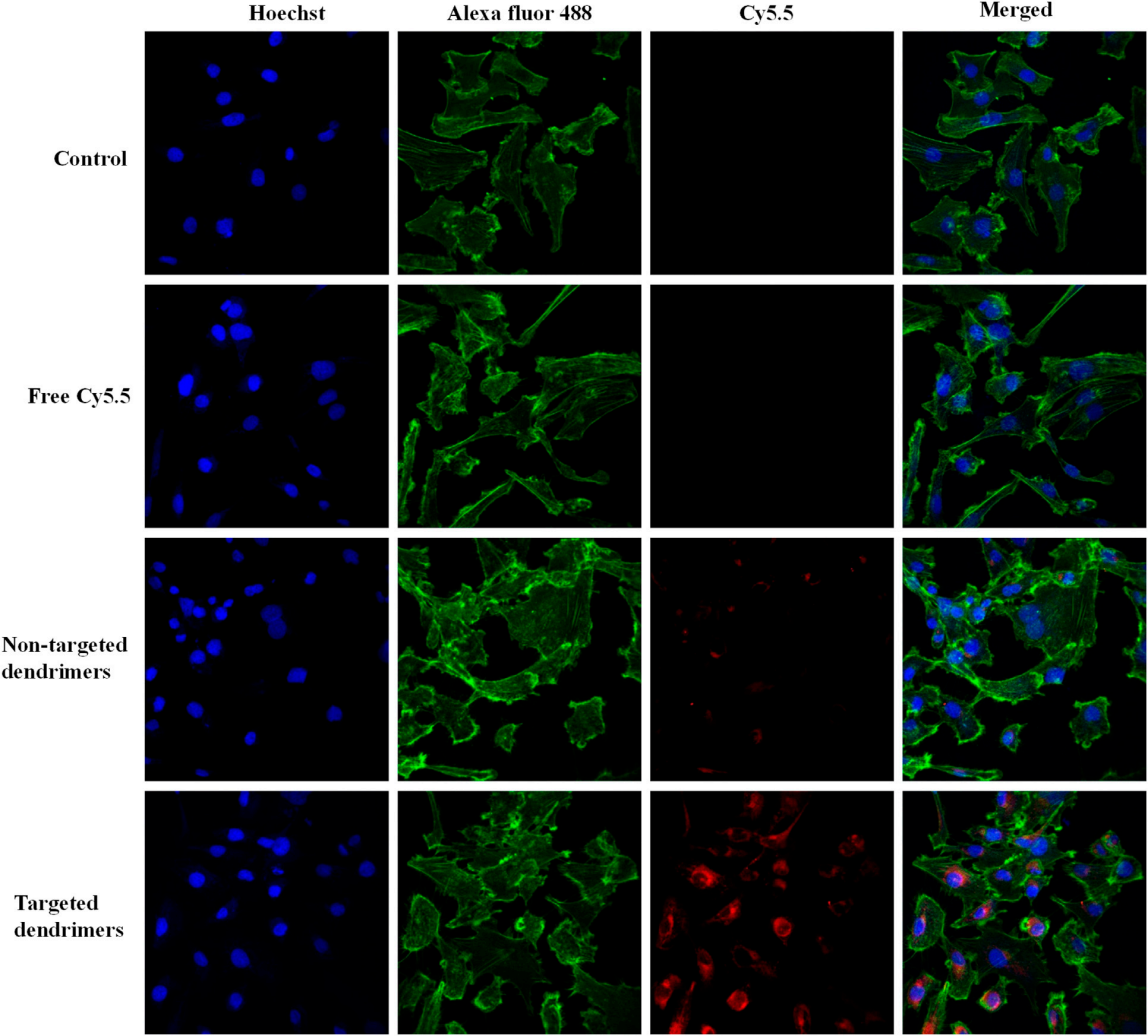
Confocal microscopy images showing comparative internalization of targeted and non-targeted NPs in MCF-7 cells.

The cellular uptake of the aptamer-conjugated targeted dendrimer was assessed against non-targeted dendrimer using fluorescence-based confocal microscopy. MCF-7 cells showed significantly higher red fluorescence in the group treated with targeted dendrimers in comparison with non-targeted dendrimers (Figure 3). Here, the higher uptake of targeted dendrimer was due to the interaction of MUC1 aptamer present over dendrimer and receptor present over MCF-7 cells. To demonstrate the specificity of MUC1 aptamer towards MCF-7 cells, the uptake of the aptamer-conjugated targeted dendrimer and non-targeted dendrimer was also assessed on MUC1 negative MDA-MB-453 cells by fluorescence-based confocal microscopy. Results of the study showed that there was very little uptake in the MDA-MB-453 cell line for both targeted and non-targeted dendrimers (Supplementary Figure S5). Further, there was no difference between targeted and non-targeted dendrimer in MDA-MB-453 cells. Here, non differentiated uptake of both the dendrimers was due to absence of MUC1 (Yamada et al., 2008). This study demonstrates the specificity of MUC1 aptamer-conjugated dendrimers for MCF-7 cells.

### 2.5 Hemolysis study

Assessing the hemolytic potential of dendrimers is widely considered advantageous in establishing their suitability for medicinal purposes. To achieve this objective, a hemolysis experiment was conducted utilizing lipofectamine-2000, targeted dendrimers, and non-targeted dendrimers. Both dendrimers showed a substantially lower hemolysis activity towards red blood cells (RBCs) compared to commercially available transfecting reagent lipofectamine-2000 (Supplementary Figure S6). This study suggests that the produced dendrimers are suitable for intravenous injection.

### 2.6 Gene silencing

**FIGURE 4:**
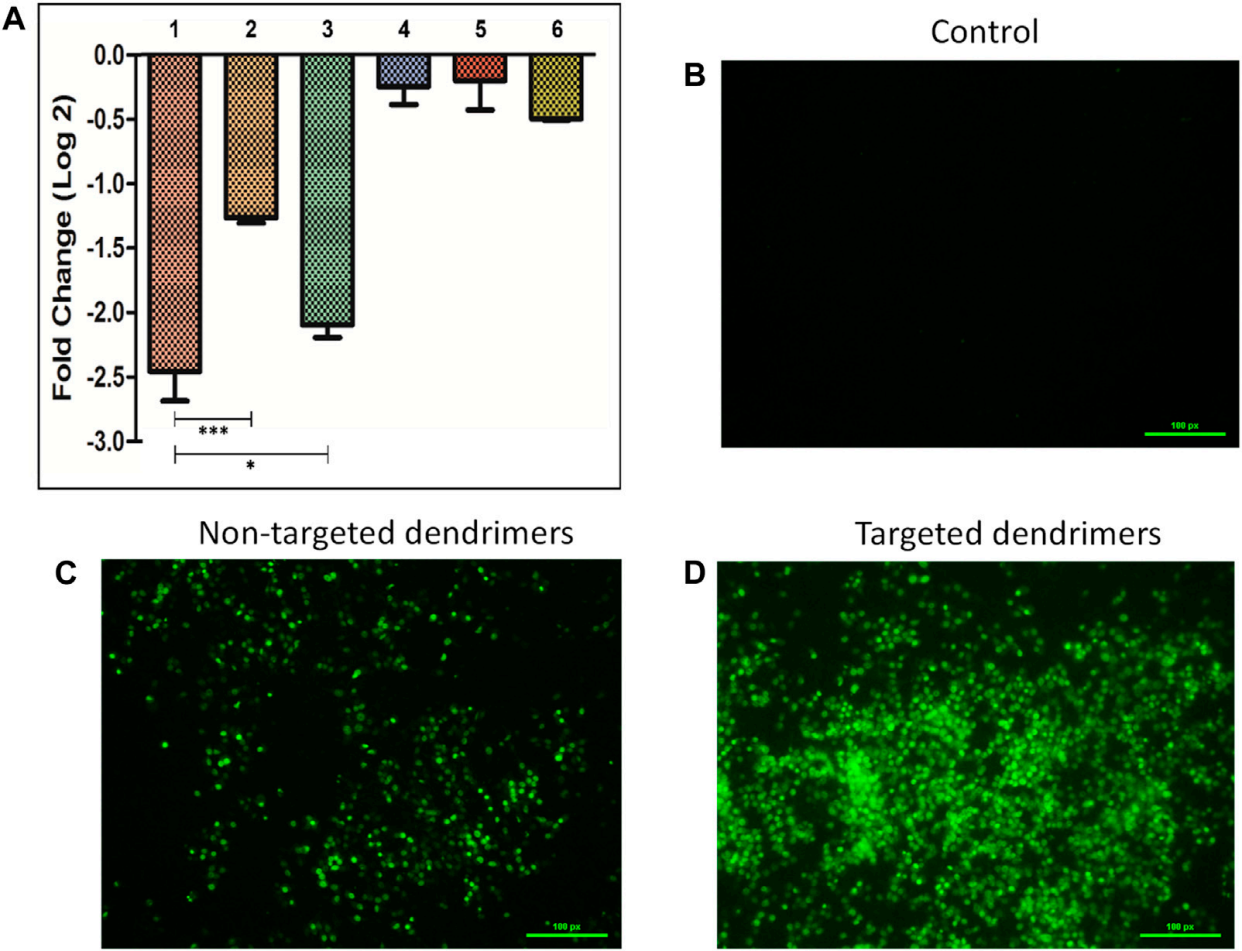
**(A)** Survivin gene silencing study in MCF-7 cells by siRNA-loaded delivery vehicles (1- Targeted dendrimer + Survivin siRNA, 2- Non-targeted dendrimer + Survivin siRNA, 3- Lipofectamine + Survivin siRNA, 4- Targeted dendrimer + Negative control siRNA, 5- Non-targeted dendrimer + Negative control siRNA, 6- Lipofectamine-2000+ Negative control siRNA). Data represented as mean ± SEM, n = 3, **p < 0.01, ***p < 0.001. Caspase activation study in MCF-7 cells **(B)** Control cells, **(C)** Cells treated with non-targeted dendrimers, and **(D)** Cells treated with targeted dendrimers.

The silencing efficacy was evaluated by transfecting MCF-7 cells with various siRNAs (Survivin and negative control siRNA) that were bound to targeted dendrimers, non-targeted dendrimers, and lipofectamine-2000 (Figure 4A). The use of targeted dendrimers for delivering siRNA into cells effectively suppressed the expression of target-specific genes. The qRT-PCR study showed that the use of targeted dendrimers for delivering siRNA led to a more significant decrease in survivin mRNA levels compared to the delivery using non-targeted dendrimers and lipofectamine-2000. The introduction of survivin siRNA-loaded targeted dendrimers into MCF-7 cells resulted in a higher silencing effect of ∼2.5-fold compared to the non-targeted dendrimers (∼1.3-fold) and lipofectamine-2000 (∼2-fold) (Figure 4A). The conjugation of TEPA and MUC1 aptamer greatly enhanced the silencing capability of the targeted dendrimers. The presence of the positively charged component TEPA has enhanced the efficacy of loading siRNA onto the dendrimers. Additionally, the MUC1 aptamer targets MCF-7 cells, hence increasing their ability to take up the dendrimers efficiently. Prior studies have demonstrated similar results where cationic moiety-modified nanocarriers were used for the siRNA delivery to suppress gene expression. Kumar et al. (2023) reported successful inhibition of the BCl-xL and BCL-2 genes in DOX-resistant MDA-MB-231 triple-negative breast cancer cells through the delivery of targeted siRNA-loaded cationic moiety-modified nanocarriers. The AS1411 aptamer binds explicitly to nucleolin, enhancing the silencing capacity of the nanocarrier modified with a cationic moiety (Kumar et al., 2023).

### 2.7 Activation of caspases and cell death

The activation of caspases was confirmed by observance of the green color inside the cells (Figures 4B–D). The cells treated with non-targeted dendrimer loaded with survivin siRNA showed lower caspase activation (lower green fluorescence) (Figure 4C). However, cells treated with targeted dendrimer loaded with survivin siRNA showed higher caspase activation (higher green fluorescence) (Figure 4D). The higher caspase activation in the targeted dendrimer group was due to efficient uptake of the dendrimer followed by higher survivin gene silencing. This study indicated that the synthesized targeted dendrimer loaded with survivin siRNA effectively activated caspases due to the downregulation of survivin inside breast cancer cells and follow apoptosis-mediated cell death.

Annexin V/PI dual staining has been used for quantitative determination of apoptosis. The increased presence of the phosphatidyl serine (PS) on the surface of plasma membrane is the key feature of apoptosis, which can be visualized by tagging with fluorescently labeled Annexin V molecules. Simultaneously, cells stained with propidium iodide (PI) enable visualization of the dead cells. The combination of both staining procedures ensures distinction among live, early apoptotic, late apoptotic and necrotic cells. Annexin V/PI dual staining study in MCF-7 cells showed that the targeted dendrimers loaded with survivin siRNA had higher cell death as compared to non-targeted dendrimers (Supplementary Figures S7, S8). At 12 h, targeted dendrimers, non-targeted dendrimers and Lipofectamine 2000 loaded with survivin siRNA showed 15.5% ± 2.64%, 6.9% ± 1.37% and 10.0% ± 1.19% apoptosis, respectively (Supplementary Figure S7). While at 48 h, significant higher cell killing was observed in cells treated with targeted dendrimers (86.2% ± 2.72% apoptosis) loaded with survivin siRNA as compared to non-targeted dendrimers (49.3% ± 3.49% apoptosis) and Lipofectamine 2000 (72.4% ± 2.29% apoptosis) (Supplementary Figure S8). Flow cytometry analysis confirmed apoptosis mediated cell death in MCF-7 cells due to silencing of survivin.

## 3 Conclusion

This research demonstrates the remarkable potential of aptamer-conjugated hyperbranched bis-MPA polyester-based dendritic nanoconjugates to deliver siRNAs precisely to MUC1 overexpressing cancer cells. With our successful synthesis and characterization of the nanoconjugate, we have shown that it can be taken up by breast cancer cells while being completely biodegradable and safe for clearance. The targeted dendrimer modified with MUC1 aptamer exhibited significantly enhanced cellular internalization and gene silencing efficacy compared to the non-targeted one. By targeting the MUC1 glycoprotein that is overexpressed in breast, lung, ovarian, and pancreatic cancer, our nanoconjugate shows immense promise for efficient cancer therapy. Furthermore, the nanoconjugate shows promise with its ability to combine with other aptamers to deliver siRNA/miRNA to specific cells and tissues, making it a highly effective tool in the bench-to-bedside translation of nucleic acid therapeutics.

## Data Availability

The original contributions presented in the study are included in the article/Supplementary Material, further inquiries can be directed to the corresponding author.
