# Supplementary material for: MUC1 aptamer-tethered H40-TEPA-PEG nanoconjugates for targeted siRNA-delivery and gene silencing in breast cancer cells
Source: Front Bioeng Biotechnol. 2024 Apr 18;12:1383495. doi: 10.3389/fbioe.2024.1383495 (PMC11063312; doi:10.3389/fbioe.2024.1383495)
Supplement: Supplementary file 1 [file DataSheet1.pdf]

MUC1 aptamer-tethered H40-TEPA-PEG nanoconjugates for targeted siRNA-delivery and gene silencing in breast cancer cells

Rajesh Salve[1,2], Niladri Haldar[1,2], Aazam Shaikh[1,2], Rajkumar Samanta[1,2], Devyani Sengar[1,2], Surajit Patra[1,2], Virendra Gajbhiye[1,2*]

[1]Nanobioscience Group, Agharkar Research Institute, Pune- 411004

[2]Savitribai Phule Pune University, Pune - 411007

Address for correspondence:

Dr. Virendra Gajbhiye

(virendragajbhiye@aripune.org; cme_virendra@yahoo.co.in)

1. Materials and Methods

1.1 Materials

Hyperbranched bis-MPA polyester dendrimer (H40-OH, generation 4), Succinic anhydride, 4-N,N-dimethylaminopyridine (DMAP), Triethylamine (TEA), Dicyclohexylcarbodiimide (DCC), N-hydroxysuccinimide (NHS), Tetraethylenepentamine (TEPA), Thi C3 modified Mucin-1 DNA aptamer (MUC1) (5'GAAGTGAAAATGACAGAACACAACA3'), Survivin siRNA (Sense strand-5'GAAAGAAUUUGAGGAAACUdTdT 3' And Anti-sense strand-5'AGUUUCCUCAAAUUCUUUC dTdT 3'), Primers (Actin, GAPDH and Survivin), Tris(2-carboxyethyl)phosphine hydrochloride (TCEP), NHS-PEG-Maleimide and NHS-PEG-OCH_3 were purchased from Sigma-Aldrich. The cell lines present in this study were obtained from National Center for Cell Sciences (NCCS), Pune, India.

1.2 Purification of H40-OH

For the purification of dendrimer, 200 mg of H40-OH was dissolved in 2 mL of acetone under continuous stirring in an inert condition and incubated overnight. After this, the resultant solution was added to chilled diethyl ether dropwise and incubated for 4 hrs at -20°C. The obtained precipitate was recovered by centrifugation at 1200 RPM for 30 mins. The pellet was dried under vacuum till complete removal of solvent (Tu *et al.*, 2013).

1.3 Synthesis of carboxylic group modified H40 dendrimer (H40-COOH)

H40-OH was converted to H40-COOH using succinic anhydride and TEA **(Figure 1)**. In detail, 110 mg of H40-OH was reacted with 140 mg of succinic anhydride in the presence of 0.03 mL of TEA. The reaction was performed in anhydrous THF (2 mL) for 24 hrs under continuous

stirring at room temperature. The obtained solution was washed with THF and diethyl ether four times (Chen *et al*., 2012).

1.4 Synthesis of TEPA-modified H40 dendrimer (H40-TEPA)

Tetraethylenepentamine was conjugated over the –COOH modified dendrimer using DCC and NHS coupling reaction **(Figure 1)**. For this, 130 mg of H40-COOH was dissolved in 5 mL of dimethyl sulfoxide (DMSO), and then 15 mg of DCC and 8 mg of NHS were added to the above solution and stirred for 12 hrs at room temperature under an inert atmosphere. Further, 30 mg of tetraethylenepentamine was added to the reaction and continued the reaction for 24 hrs. Upon completion of the reaction, the resultant solution was filtered to remove the by-product, i.e., dicyclohexylurea (DCU). Subsequently, the obtained product was dialyzed against DMSO for 24 hrs and against distilled water for 24 hrs using a 2 kDa molecular weight cut-off dialysis membrane to remove impurities (Chen *et al*., 2019).

1.5 Synthesis of targeted (H40-TEPA-PEG-MUC1) and non-targeted (H40-TEPA-PEG-OCH$_3$) dendrimers

Targeted and non-targeted dendrimers were synthesized by following the below steps (**Figure 1**). For the synthesis of the targeted dendrimers, five molecules of PEG and MUC1 aptamer were conjugated over each dendrimer. For this, H40-TEPA was dissolved in DMSO and allowed to react with NHS-PEG-maleimide for 24 hrs in an inert atmosphere under continuous stirring. Further, MUC1 aptamer treated with TCEP in water was added to the above reaction and allowed to continue the reaction for 24 hrs. After completion of the reaction, the above solution

is dialyzed against water and lyophilized. Similarly, in the case of non-targeted dendrimer synthesis, NHS-PEG-OCH_3 was reacted with H40-TEPA (Li *et al*., 2012).

1.6 Characterization of dendrimer

^{1}H NMR was carried out to confirm synthesis of H40-COOH, H40-TEPA, H40-TEPA-PEG and H40-TEPA-PEG-MUC1. The ^{1}H NMR spectra of the samples were obtained by using a Bruker Avance III HD NMR 500 MHz spectrometer, and DMSO (d6) was used as the solvent. The size and zeta potential of both H40-TEPA-PEG-MUC1 and H40-TEPA-PEG-OCH_3 dendrimers were determined by dynamic light scattering (Malvern Zetasizer ZSP, Malvern, Germany).

1.7 Gel retardation assay

This assay was performed to assess the binding efficiency of targeted dendrimers with siRNA using agarose gel electrophoresis. The process of complexation between siRNA and dendrimers was carried out at different N/P ratios (N/P 5:1, 10:1, 15:1, 20:1, 25:1, and 50:1). Complexes were developed by incubating dendrimers with siRNA at room temperature for 1 hr and evaluated by performing gel electrophoresis using a 3% agarose gel at 65 mV. Free siRNA was employed as a negative control (Tambe *et al*., 2017).

1.8 Cell viability assay

The MTT assay was used to determine the possible cytotoxicity of the synthesized dendrimers. For this, NIH-3T3 cells were seeded at a density of $1x10^4$ cells per well in a 96-well plate and incubated at 37°C with 5% CO_2. After 24 hrs, the medium was removed, and the cells were

treated with targeted NPs at different concentrations (25, 50, 75, 100, and 150 μg/mL) for 48 hrs in incomplete medium. Then, 10 μL of MTT reagent (5 mg/mL) was added to each well and incubated for 4 hrs in the CO_2 incubator. Formed MTT formazan crystals in the wells were dissolved in 200 μL of DMSO. The absorbance was measured at 570 nm using the BioTek Synergy plate reader (Yu *et al*., 2014).

1.9 Cellular uptake visualization using confocal microscopy

In a 6-well plate, 1×10^5 MCF-7 or MDA-MB-453 cells were seeded per well and incubated at 37℃ for 24 hrs. The cells were then incubated with fluorescently tagged dendrimer (100µg/mL) for 2 hrs in serum-free media. The treatment was ended by removing the media and washing the cells with PBS. The cells were then fixed, permeabilized, stained, and imaged using a confocal microscope (Leica Microsystems, Model TCS-SP8, Germany) (Liu *et al*., 2015).

1.10 Hemolysis Study

The purchased EDTA stabilized sheep blood was diluted (1:1) in PBS and centrifuged at 3000 rpm for 5 min. The red blood cells (RBCs) in the pellet were dispersed in PBS, and this step was repeated three times. Further, obtained RBCs were diluted in a glucose solution (5% w/v). RBCs were treated with appropriately diluted targeted dendrimers, non-targeted dendrimers, and lipofectamine 2000. Triton-X 100 was used as a positive control, and PBS as a negative control. The mixture was incubated at 37˚C for 2 hrs and then centrifuged to settle the cells. The absorbance of the supernatant was measured at 540 nm. The % hemolysis was calculated, and the graph was plotted (Vankoten *et al*., 2016).

1.11 Gene silencing

The gene silencing study was performed to check the silencing ability of synthesized dendrimers in MCF-7 breast cancer cells. For this study, MCF-7 cells were seeded on a 24-well plate at 10^5 cells/well density. Treatment was initiated once cells were attached to the substratum. The survivin gene silencing efficacy was investigated by administering a 100 nM siRNA for a duration of 24 hrs. Survivin siRNA complexed with targeted dendrimers (N/P ratio 25:1), non-targeted dendrimers (N/P ratio 20:1), and lipofectamine-2000 was added to cells in Opti-MEM media. Total RNA was extracted from the cell samples and then complementary DNA (cDNA) was prepared. Quantitative real-time PCR (qRT-PCR) (Light Cycler 480, Roche, Germany) was then used to investigate survivin gene expression levels in different treatment groups. The sequence of primers is given in table 1. To calculate the ΔCt value of the target gene for every group, the β-actin, and GAPDH gene was used as an internal control. The following formula was used to find the relative expression of the survivin gene:

Relative quantification (RQ) = $2^{-\Delta\Delta Ct}$

For the gene-silencing analysis, the transfecting agent, lipofectamine 2000 (ThermoFisher Scientific, 11668019), was used as the positive control, whereas the negative control siRNAs (Merck, SIC001-10NMOL) complexed with targeted nanocarriers, non-targeted nanocarriers, and lipofectamine-2000 was used as a negative control (Kumar *et al*., 2023).

1.12 Caspase assay:

Caspase-3/7 Green Detection Reagent (Molecular probes, Invitrogen, USA) was used to confirm caspase-3 and caspase-7 activation in MCF-7 cells. In detail, MCF-7 cells ($1x10^5$) were treated

with survivin siRNA loaded with non-targeted dendrimer and survivin siRNA loaded with targeted dendrimer for 24 hrs in an incomplete medium. Then, caspase-3/7 Green Detection Reagent was added to the treated cells and incubated for 1 hr. Then the cells were visualized under a fluroscence microscope (Nikon Eclipse TS 100, Japan) (Killinger *et al*., 2021).

1.13 Flow cyometry by Annexin V/PI staining

$1x10^5$ MCF-7 cells were seeded and grown overnight in 24 well plates at 37°C in CO_2 incubator. The cells were then treated with non-targeted dendrimer, targeted dendrimer and Lipofectamine 2000 complex with the Survivin siRNA. After 12 and 48 hrs, cells were harvested and centrifuged (1000 RPM, 10 mins) to remove media residues. Cells were resuspended in 100 µL Annexin binding buffer. Thereafter, cells were stained with Alexa Fluor 488 Annexin V and propidium iodide (FITC annexin V/dead cell apoptosis kit, molecular probe) for 15 min at room temperature. The volume of the suspension was made up to 500 µL with annexin binding buffer. Samples were analyzed by flow cytometer (BD FACS Calibur, BD Biosciences, USA) (Crowley *et al*., 2016).

1.14 Statistical analysis

Each experiment was performed in triplicate, data presented as means ± standard deviation (SD). Statistical analysis was carried out by using GraphPad Prism software. The Statistical significance was checked by two-way analysis of variance (ANOVA) with Bonferroni posttests. P value <0.05 was found significant.

Figures and Tables

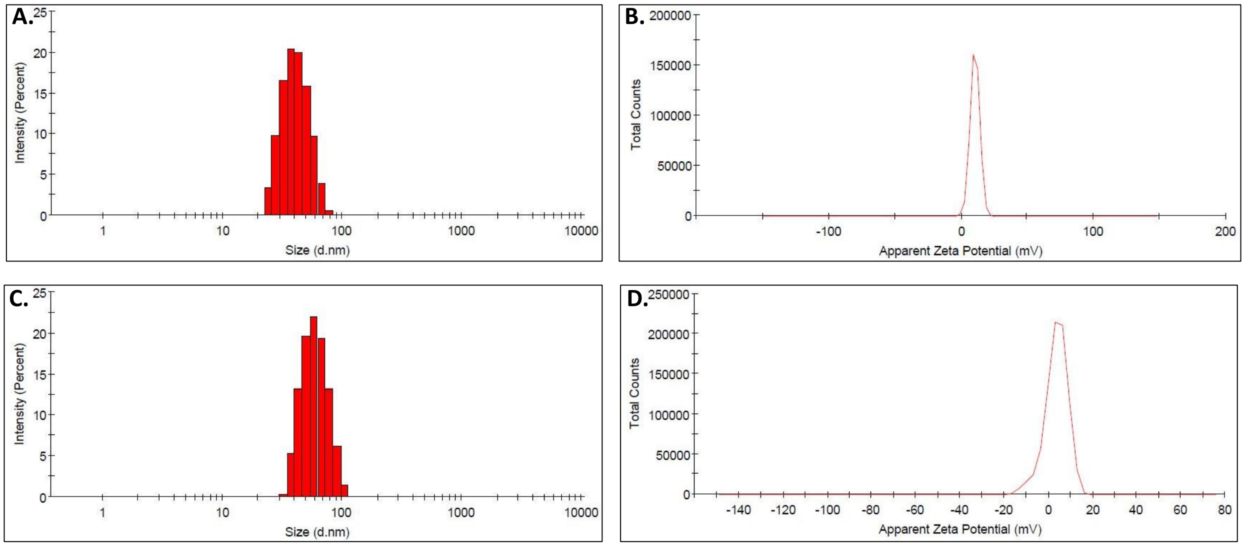

Supplementary Figure S1: Hydrodynamic size and zeta potential of non-targeted dendrimers (A&B) and targeted dendrimers (C&D).

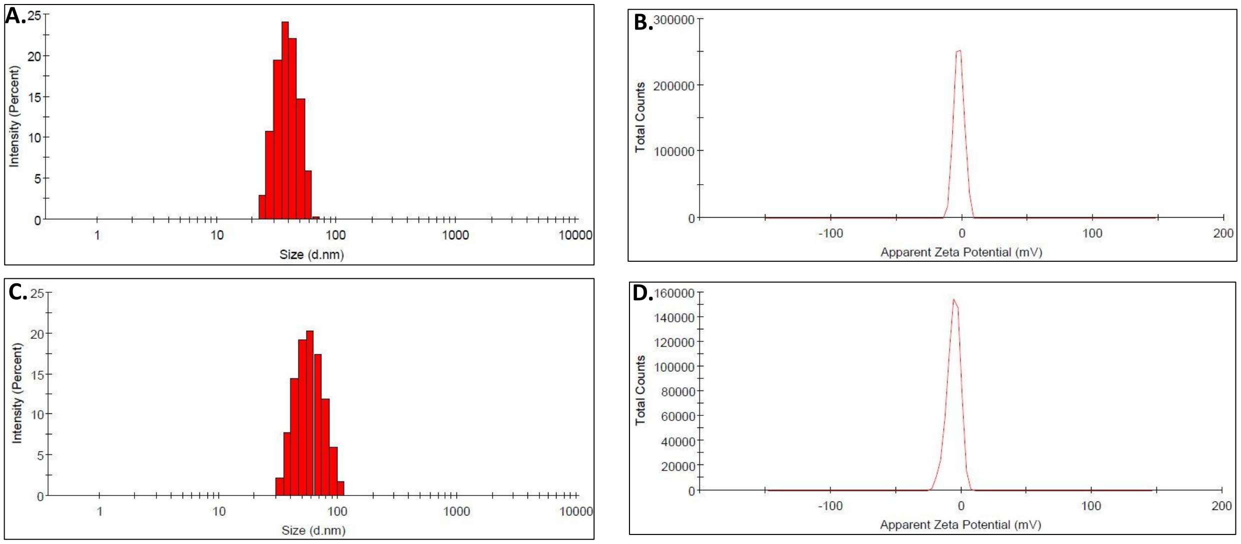

Supplementary Figure S2: Hydrodynamic size and zeta potential of siRNA loaded non-targeted dendrimers (A&B) and targeted dendrimers (C&D).

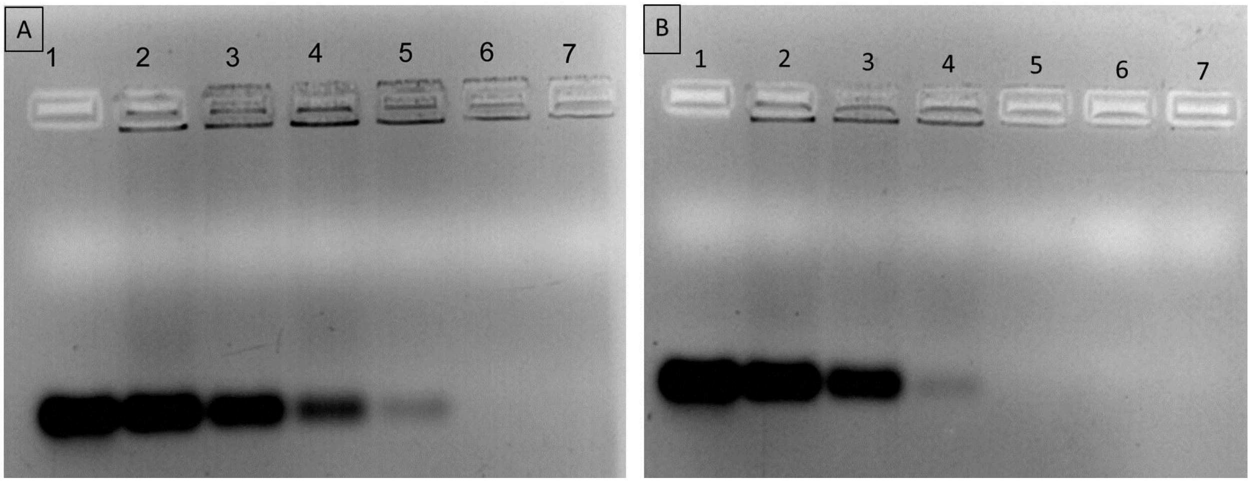

Supplementary Figure S3: Agarose gel electrophoresis studies for complexation of siRNA with targeted dendrimers and non-targeted dendrimers. Lane 1: siRNA control, lane 2-7: NPs/siRNA complexes (N/P ratios 5:1, 10:1, 15:1, 20:1, 25:1 and 50:1).

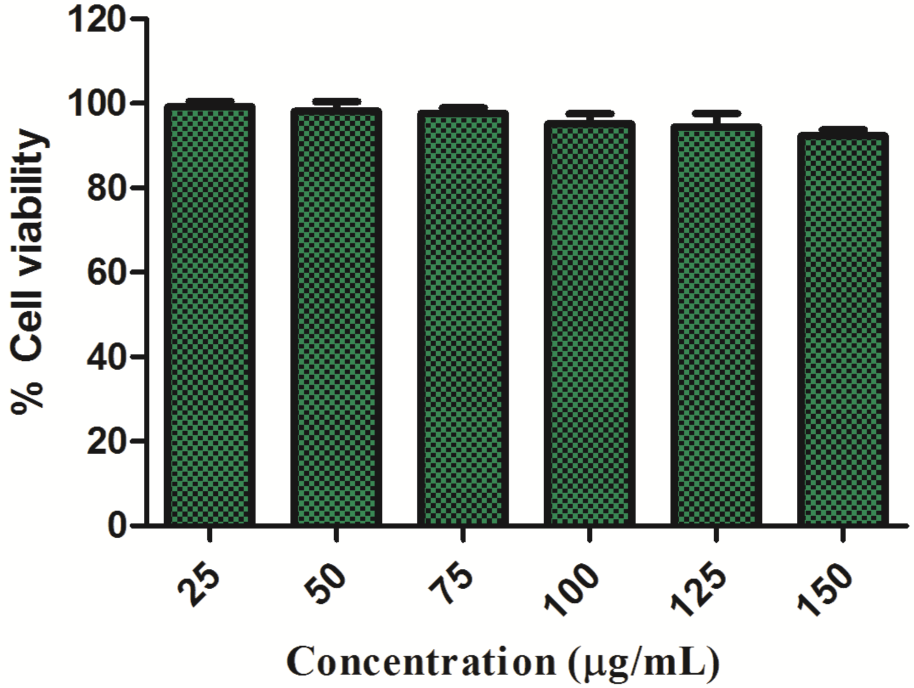

Supplementary Figure S4: Cytotoxicity study of targeted dendrimers by MTT assay on NIH-3T3 cells.

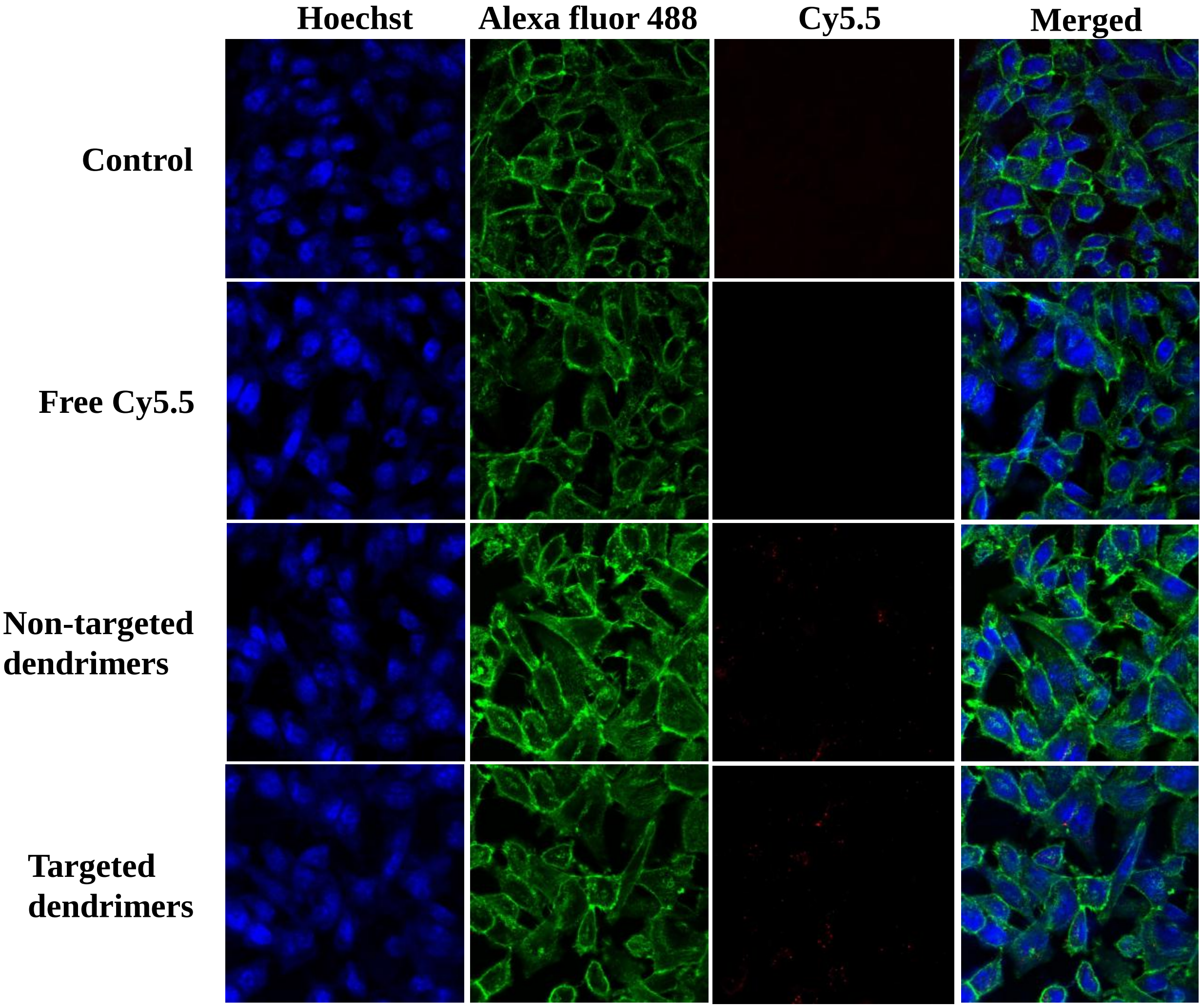

Supplementary Figure S5. Confocal microscopy images showing internalization of targeted and non-targeted NPs in MUC-1 negative MDA-MB-453 cells.

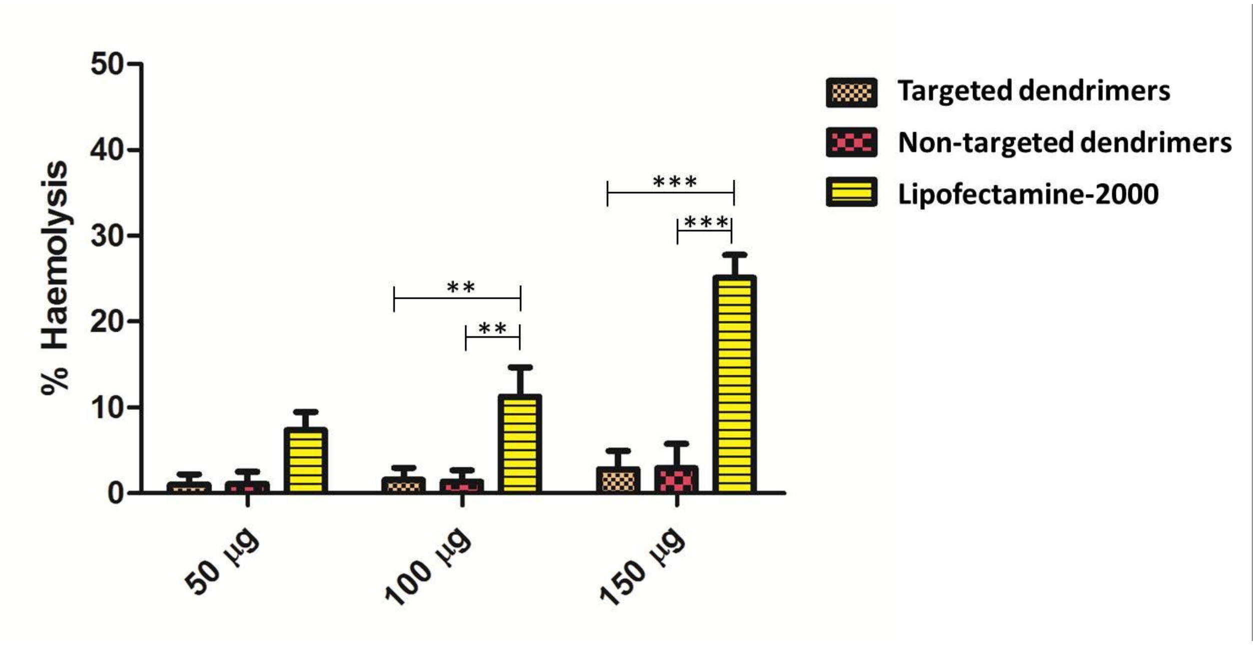

Supplementary Figure S6: Hemocompatibility study of the synthesized targeted and non-targeted dendrimers.

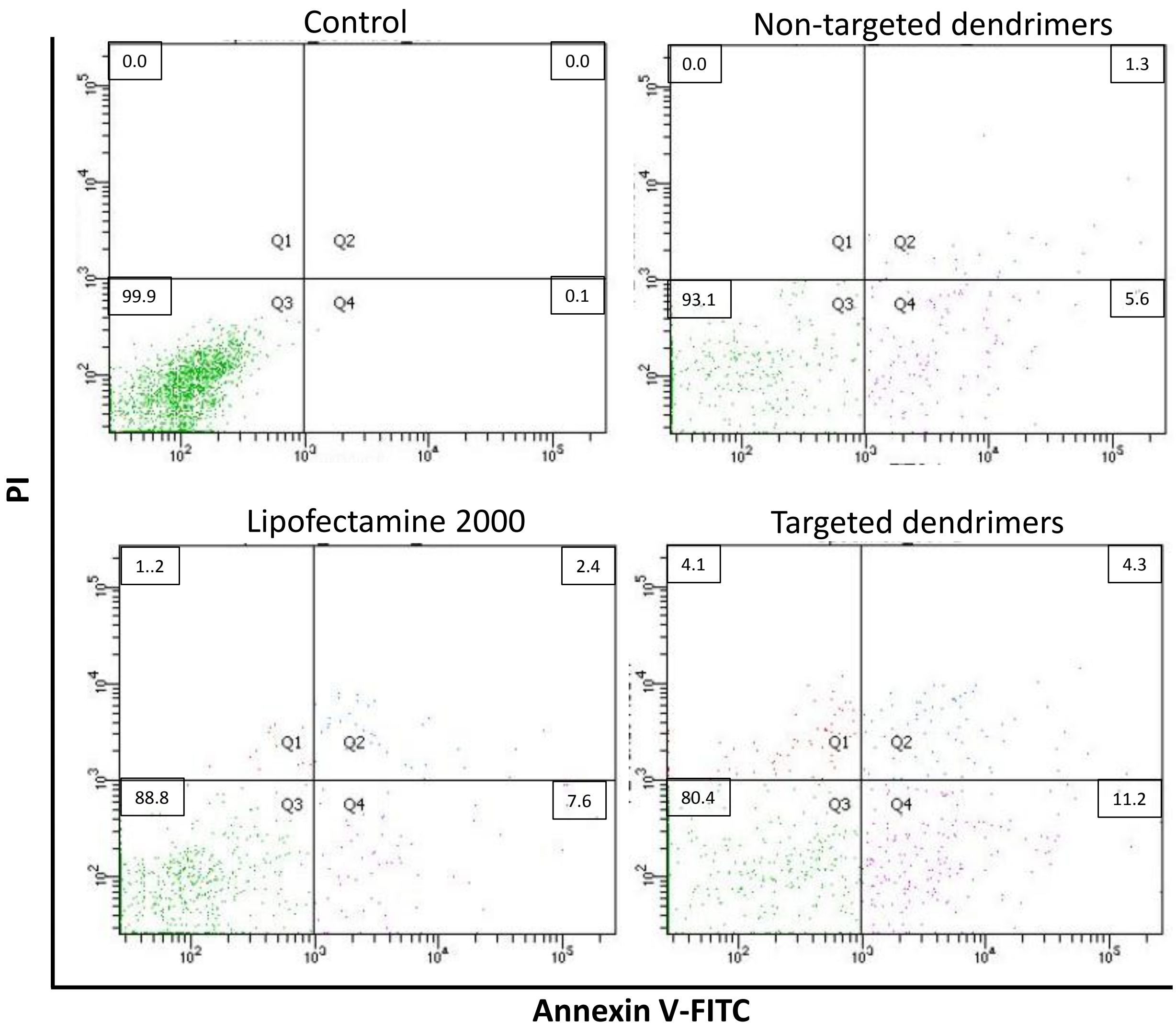

Supplementary Figure S7: Flow cytometry analysis of Annexin V/PI stained, siRNA treated MCF-7 cells at 12 hr.

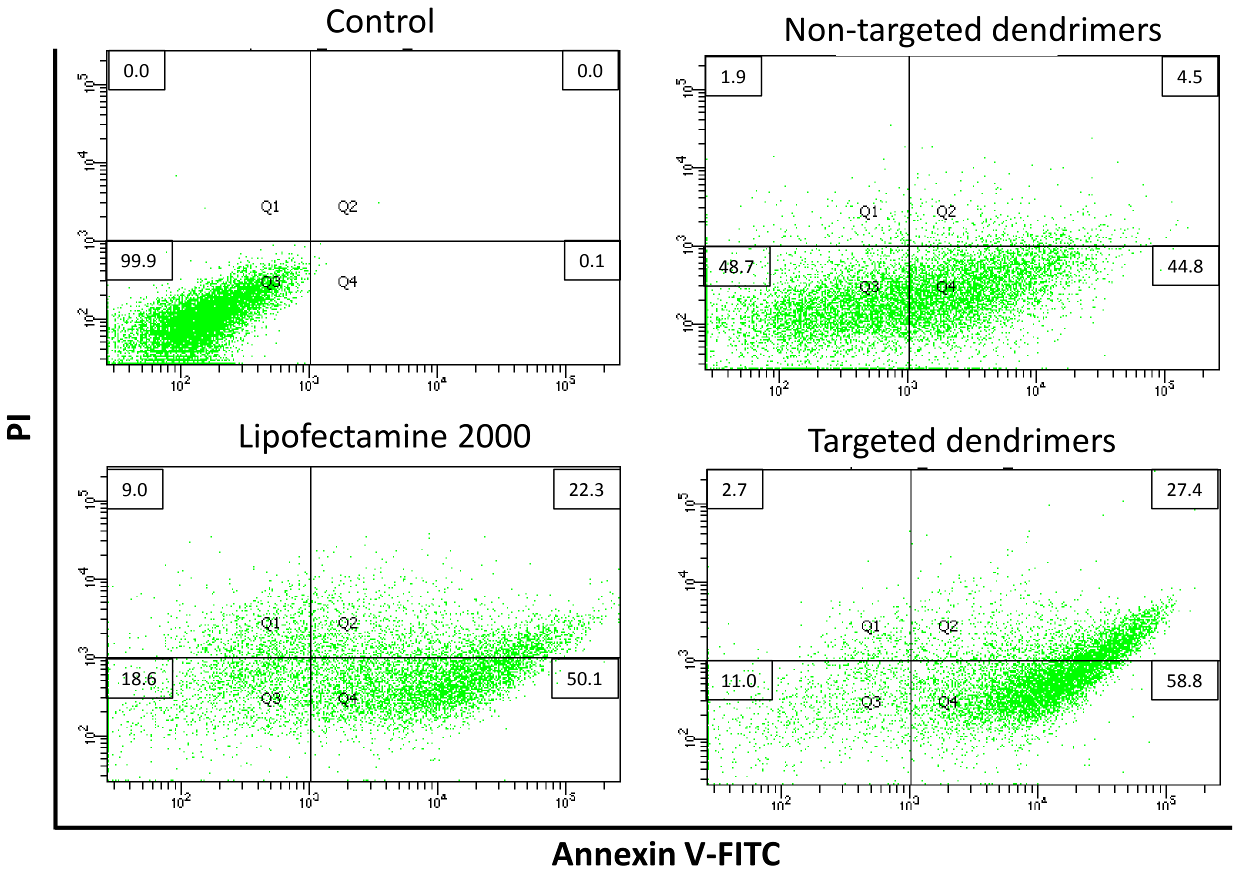

Supplementary Figure S8: Flow cytometry analysis of Annexin V/PI stained, siRNA treated MCF-7 cells at 48 hr.

Table 1. Primer sequences used for real-time PCR.

Sr. No.	Gene	Primer Sequence
1	Survivin	Forward: 5'CTGCTTCTCTCTCTCTCTCTCT<br><br>Reverse: 5'ACATGAGGTCCAGACACATTC
2	Actin	Forward: 5'CACCAACTGGGACGACAT<br>Reverse: 5'ACAGCCTGGATAGCAACG
3	GAPDH	Forward: 5'GCTCCCTCTTTCTTTGCAGC<br>Reverse: 5'CCCAGCTCTCATACCATGAGTC
